# Fabrication of Large-Aspect-Ratio Micro Tool Electrodes by Bipolar Pulsed Vertical Liquid Membrane Method

**DOI:** 10.3390/mi16060636

**Published:** 2025-05-28

**Authors:** Xiujuan Wu, Li Wang, Weijing Kong, Tao Yang, Yusen Hang, Yongbin Zeng

**Affiliations:** 1 College of Mechanical Engineering, Nanjing Vocational University of Industry Technology, Nanjing 210023, China; kongwj@niit.edu.cn (W.K.); yangtao860632@163.com (T.Y.); 2023101416@niit.edu.cn (Y.H.); 2 Engineering Research Centre for Industrial Sensing and Intelligent Manufacturing Equipment of Jiangsu Province, Nanjing 210023, China; 3 College of Mechanical and Electrical Engineering, Nanjing University of Aeronautics and Astronautics, Nanjing 210016, China; lwang05109@nuaa.edu.cn (L.W.); binyz@nuaa.edu.cn (Y.Z.); 4 Nanjing Institute of Astronomical Optics & Technology, Chinese Academy of Sciences, Nanjing 210042, China

**Keywords:** bipolar pulse, vertical liquid membrane, insoluble electrolytic products, micro tool electrode

## Abstract

To achieve efficient preparation of microfine tool electrodes with a large aspect ratio, a bipolar pulse vertical liquid membrane electrochemical etching technique was proposed. The difference in current density distribution on the surface of tungsten rods under single-ended and double-ended vertical liquid membrane methods was analyzed using COMSOL software. The effects of negative voltage and pulse width on the distribution of electrolytic products and electrode preparation were investigated. It was found that when a large number of hydrogen bubbles were generated on the surface of the electrode, the electrode lost the protection of the diffusion layer, and the length was drastically shortened. When the pulse width was large, the electrode surface was covered with a coating layer of insoluble electrolysis product, and the shortening of electrode length was suppressed. Subsequently, the effects of forward voltage and bias on electrode preparation were investigated for large pulse widths. The optimal parameters are as follows: electrolyte concentration of 0.5 M, forward voltage of 4 V, negative voltage of −2 V, pulse period of 50 microseconds, and pulse width of 40 microseconds. Finally, the tool electrode with an average diameter of about 23.8 μm and an aspect ratio of 91.2 was prepared.

## 1. Introduction

With the rapid development of aerospace, electronics, medical testing and communications, and other fields, products are increasingly becoming miniaturized and intelligent [1,2,3], with micro-miniature products playing an increasingly important role. The micro-machining technologies at present include micro-cutting, electrochemical micro-machining, electrical discharge micro-machining, and so on. Micro tools are one of the key factors in micro-machining, and their materials, dimensions, and shapes affect the machining results of microstructures. In particular, when machining critical features such as deep holes or deep grooves, the electrode not only needs to be small in size but also needs to have an ultra-large aspect ratio. Therefore, “micro tool with ultra-large aspect ratio” for processing micro-miniature products have received widespread attention. Ali et al. [4] successfully prepared a 90 μm long micro milling cutter with an aspect ratio of 4.5 by combining wire EDM (Electrical Discharge Machining) and focused ion beam milling techniques. Xu et al. [5] prepared a cylindrical electrode with a large length-to-diameter ratio of 310 by using ultrasonic vibration-assisted centerless grinding with distributed operations. Wang et al. [6,7] proposed a double-mirror line tangential feed electrical discharge grinding method to address the conical issue of the electrode during machining and successfully fabricated a micro-electrode with a diameter of 39.38 µm and a length-to-diameter ratio greater than 50. Although the aforementioned methods for fabricating micro-electrodes can achieve the production of tool electrodes with a large aspect ratio, they have many shortcomings compared with micro-electrochemical machining technology. These methods require complex machining equipment, incur higher processing costs, and have lower processing efficiency. In addition, the electrode surfaces produced by these methods often suffer from issues such as recast layers.

Micro-electrochemical machining (MECM) typically involves a cathode tool, an anode workpiece, and an electrolyte. The shape and dimensions of the tool electrode have a direct impact on the machining accuracy, size, and stability of the final machining result [8]. Compared with other micro-fabrication technologies, micro-fabrication electrochemical machining has received widespread attention because of its advantages of non-contact machining, being unrestricted by the mechanical properties of the material, and good quality of the machined surface [9,10]. Electrochemical etching techniques mainly include the immersion method and liquid membrane method.

When electrodes are prepared by the immersion method, the cathode is usually round-walled and circular, and the anode passes through the center of the cathode tool and is immersed in the electrolyte. Under the action of electrochemical corrosion, the anode electrode is gradually thinned and forms a cone or column tool electrode. Kamaraj et al. [11] successfully fabricated a high-aspect-ratio metal electrode with a length of 2200 µm and an average diameter of 6 µm by rotating the anode. Patro et al. [12] used high-carbon steel as the tool electrode material, analyzed the role of the black product film on the surface, and fabricated a tool electrode with an average diameter of 80 µm and an aspect ratio greater than 75. In addition, to enhance the controllability of the electrode fabrication process, Peng et al. [13] proposed an online fabrication method for micro-electrodes based on critical reaction distance control and successfully fabricated a micro-electrode with a diameter of 10 µm and an aspect ratio greater than 200. Xiong et al. [14] investigated the effect of electrode rotation on the flow field near the electrode based on the theory of spiral vortex in the Taylor–Couette system. The effects of rotation parameters and other process parameters on the thread shape of the electrode were also analyzed. Although the immersion method has some advantages in preparing tool electrodes with large aspect ratios, the electrode is always connected to the circuit during processing, the diameter of the electrode tip is greatly affected by the power supply disconnection time, and the electrode is prone to fracture at the interface of the liquid membrane and air.

In liquid membrane electrochemical etching technology, a metal ring is used as the cathode, and the electrolyte is suspended on the metal ring by the surface tension of the liquid. The electrode falls naturally due to gravity when the preparation is finished, which breaks the circuit; the phenomenon of electrode passivation caused by system delay can be avoided, and the electrode limit size is small. However, the electrolytic products are affected by gravity and the shape of the liquid membrane, resulting in the formation of an inverted cone-shaped diffusion layer around the lower electrode, which in turn leads to the existence of a certain taper in the prepared electrodes. Ali et al. [15] conducted comparative tests for the preparation of needle tips by alternating current (AC) etching, meniscus etching and direct current (DC) etching techniques for 0.4 mm tungsten wires. The effect of different voltages and NaOH solution concentrations on the taper angle and diameter of the produced tips was investigated. Wang et al. [16] proposed weakening the effect of the diffusion layer by anode vibration and prepared a nano-electrode with an average diameter of 320 nm and an aspect ratio of 60. Ao et al. [17] from Tianjin University used the density difference between different solutions to form a membrane of electrolytes above the carbon tetrachloride and applied a linear reciprocating motion to a tungsten rod. The dissolution of the tungsten rod occurred only in the electrolyte membrane region, and a micro tool with an average diameter of 24.3 μm and an aspect ratio of 195.47 was created, which had a reverse conical tip. Sang et al. [18,19] adopted the vertical liquid film method to force the machining products to move downward under the action of gravity and away from the machining area. Under the condition of DC power supply, they prepared a micro-tool with an average diameter of 12 μm and a length of 700 μm, representing a large aspect ratio.

In the above-mentioned electrode preparation processes, measures such as anode vibration, reciprocating motion, and optimizing the electrode placement can, to some extent, weaken the influence of the diffusion layer, thereby increasing the aspect ratio of the electrode and reducing the tip radius. However, in order to maintain the stability of the machining environment, the amplitude of these motions is usually small, and their impact on the electrode shape is limited. Wu et al. [20] prepared electrodes of different shapes by controlling the diffusion layer but did not prepare electrodes with a large aspect ratio.

In this paper, a bipolar pulsed vertical liquid membrane method is proposed for the preparation of microfine tool electrodes. Insoluble electrolytic products will be generated on the electrode surface by using the bipolar pulsed method, which determines the morphology of the electrode. The effects of negative voltage, pulse width, positive voltage, and electrode bias on the distribution of electrolytic products, as well as the electrode preparation, are investigated. By adjusting the processing parameters, the electrode surface was uniformly covered with an insoluble electrolytic product layer, and then the tool electrode with large aspect ratio was prepared.

## 2. Principle of the Proposed Method

### 2.1. Fundamental Principle

**Figure 1:**
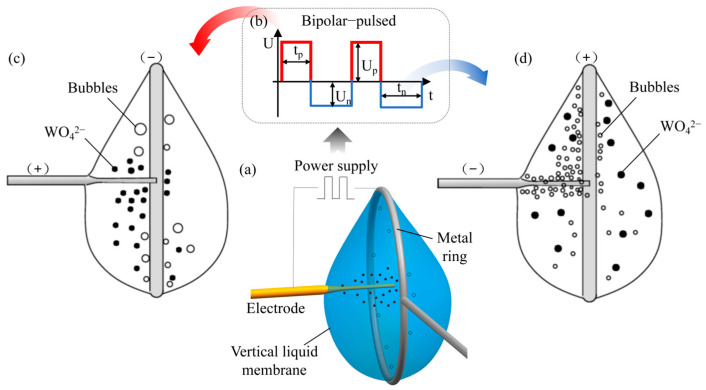
Schematic of bipolar pulsed vertical liquid membrane electrochemical etching: (**a**) Principle of bipolar pulsed vertical liquid membrane processing, (**b**) Schematic diagram of bidirectional pulse power supply, (**c**) Schematic diagram of positive pulse stage processing and (**d**) Schematic diagram of negative pulse stage processing.

The schematic diagram of the bipolar pulsed vertical liquid membrane method is shown in Figure 1a. A straight tungsten rod of 99.9% purity and a 200 μm diameter was used as the anode. A platinum wire with a diameter of 300 μm was selected, and it was wound to form a cathode ring with a diameter of 5 mm. KOH solution was used as the electrolyte. The electrolyte was attached to the platinum wire ring, and a vertical liquid membrane with a narrow top and a wide bottom was formed under the surface tension and gravity of the electrolyte. A straightened tungsten rod was threaded into the liquid membrane from the center of the ring. During the process, a bi-directional pulse power supply was applied between the straightened tungsten rod and the metal ring as shown in Figure 1b, where $U_{p}$ denotes the size of the positive voltage, $U_{n}$ denotes the size of the negative voltage, $t_{p}$ denotes the positive pulse time, and $t_{n}$ denotes the negative pulse time. After the power supply was turned on, in the positive pulse stage, the straightened tungsten rod was connected to the positive pole of the power supply, and the metal ring was connected to the negative pole of the power supply. At this time, the tungsten rod was electrolytically etched, and the following reactions occurred in the liquid membrane:

Anode reaction:

(1) $$\begin{array}{c} W\left(s\right)+6{OH}^{-}\to WO_{3}\left(s\right)+{3H}_{2}O+6e^{-} \end{array}$$

(2) $$\begin{array}{c} WO_{3}\left(s\right)+2{OH}^{-}\to {WO}_{4}^{2-}+H_{2}O \end{array}$$

Cathode reaction:

(3) $$\begin{array}{c} 6H_{2}O+6e^{-}\to 3H_{2}\left(g\right)+6{OH}^{-} \end{array}$$

The overall reaction:

(4) $$\begin{array}{c} W\left(s\right)+2{OH}^{-}+2H_{2}O\to WO_{4}^{2-}+3H_{2}\left(g\right) \end{array}$$

The electrolytic products generated near the tungsten rod moved away from the electrode under the effect of gravity and concentration gradient. At the same time, the hydrogen bubbles generated on the metal ring moved upward under buoyancy force and disturbed the electrolytic products, as shown in Figure 1c. In the negative pulse stage, the straightened tungsten rod was connected to the negative pole of the power supply, and the metal ring was connected to the positive pole of the power supply. At this stage, no etching reaction occurred on the surface of the metal ring. Meanwhile, a hydrogen precipitation reaction occurred on the tungsten rod, generating a large number of hydrogen bubbles, which further dispersed the products accumulated on the surface of the electrode, as shown in Figure 1d. By continuously applying a bipolar pulsed current, the electrode of the desired diameter could be obtained after a period of processing.

### 2.2. Effect of Negative Voltage on the Motion Process of Electrolytic Products

**Figure 2:**
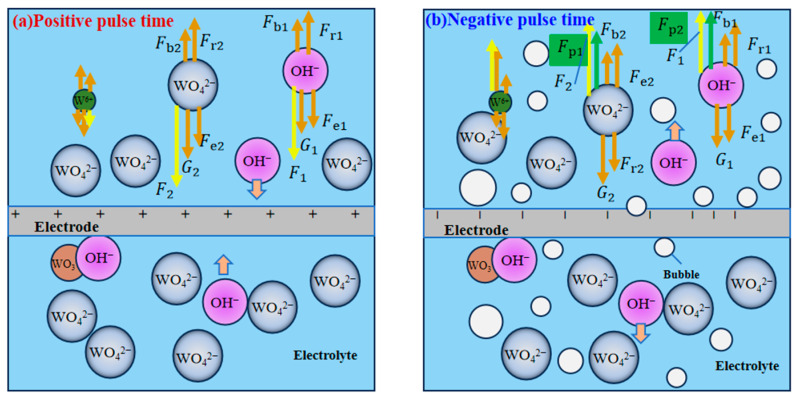
Force analysis diagram of electrolysis products.

In the traditional unidirectional pulse liquid membrane process, the electrolytic products, metal ions, and anions inside the liquid membrane are subjected to their own gravity G, buoyancy $F_{b}$, electric field force $F_{e}$, solution resistance $F_{r}$, combined force on the ${OH}^{-}$ $F_{1}$ and combined force on the ${WO}_{4}^{2-}$ $F_{2}$, as shown in Figure 2a. Since the electric field force is always greater than the solution resistance, the ${OH}^{-}$ ions in the solution keep moving towards the tungsten rod, and then electrochemical reactions occur on the surface. The ${WO}_{4}^{2-}$ ions in the solution also move towards the tungsten rod under the action of combined force and move downward slowly along the surface of the tungsten rod. In the pulse interval stage, the products and ions under the effect of inertia first make a decelerating motion and then diffuse into the solution. During the next pulse-on time, the products within the liquid membrane repeat the above motion and finally form an inverted cone-shaped diffusion layer within the liquid membrane.

When the processing is carried out with a bipolar pulsed power supply, in the positive pulse stage, the force on each product in the liquid membrane is consistent with that in the unidirectional pulse liquid membrane method of processing. However, when processing enters the negative pulse stage, the products are subjected to the reverse electric field force. As a result, the anions within the liquid membrane move in the direction away from the tungsten rod. In addition, during the negative pulse stage, a hydrogen precipitation reaction occurs on the surface of the tungsten rod, which generates many hydrogen bubbles. These bubbles not only disperse the diffusion layer attached to the surface of the tungsten rod during the positive pulse phase but also generate an additional driving force on the anions in the solution $F_{p}$. This driving force caused by bubble perturbation forces the electrolytic products to accelerate away from the tungsten rod. When the driving force $F_{p}$ is sufficiently large, it may even cause the anion to travel a much greater distance in the negative pulse phase than in the positive pulse phase. As a result, the ${WO}_{4}^{2-}$ and ${OH}^{-}$ ions are progressively driven away from the electrode surface throughout the processing stage, leading to a significant reduction in the concentration of electrolyte near the electrode. Consequently, the electrochemical reactions become difficult to sustain, thereby hindering the preparation of the electrode. By adjusting the appropriate electrical parameters, the effect of the diffusion layer on the morphology of the tool electrode can be avoided, keeping the electrode surface clean and improving the aspect ratio and processing efficiency of the electrode.

## 3. Experimental Setup

**Figure 3:**
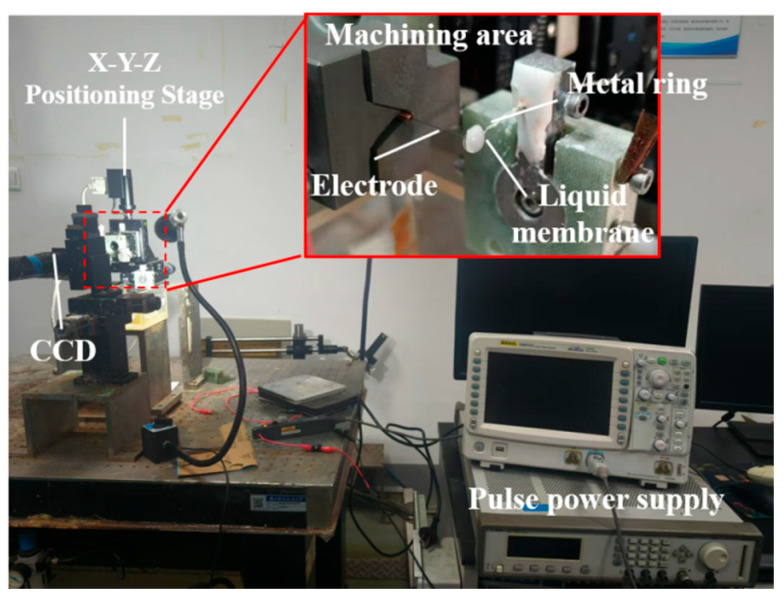
Schematic of the experimental setup used for bipolar pulsed vertical liquid membrane electrochemical etching.

Figure 3 shows the device diagram of the bipolar pulsed vertical liquid membrane method for the preparation of microfine tool electrodes. The device mainly includes XYZ axis and a motion control system, pulse power supply, a CCD real-time observation system, processing fixture, electrolyte tank, and so on. The resolution of the motion control system is 0.1 μm. The tool electrode is a tungsten rod with a diameter of 0.2 mm and a purity of 99.99%. The tungsten rod is fixed in a copper rod with a crosscut and is fixed to the electrode fixture by means of V-groove positioning, finally connected to the *Z*-axis. A CCD observation system was used to adjust the relative position of the electrode to the liquid membrane. In order to avoid the negative pulse stage, the metal ring was dissolved and destroyed, the inert metal material platinum wire was selected as the metal ring material, and the diameter of platinum wire was 0.3 mm. KOH was selected as the electrolyte. In order to ensure the stability of electrolytic processing, the thickness of the liquid membrane is controlled at 3~4 mm. During the process, the electrolyte is renewed at the same time interval, and the thickness of the membrane remains unchanged before and after the renewal of the electrolyte is controlled by the CCD system.

## 4. Results and Discussion

### 4.1. Electric Field Simulation

**Figure 4:**
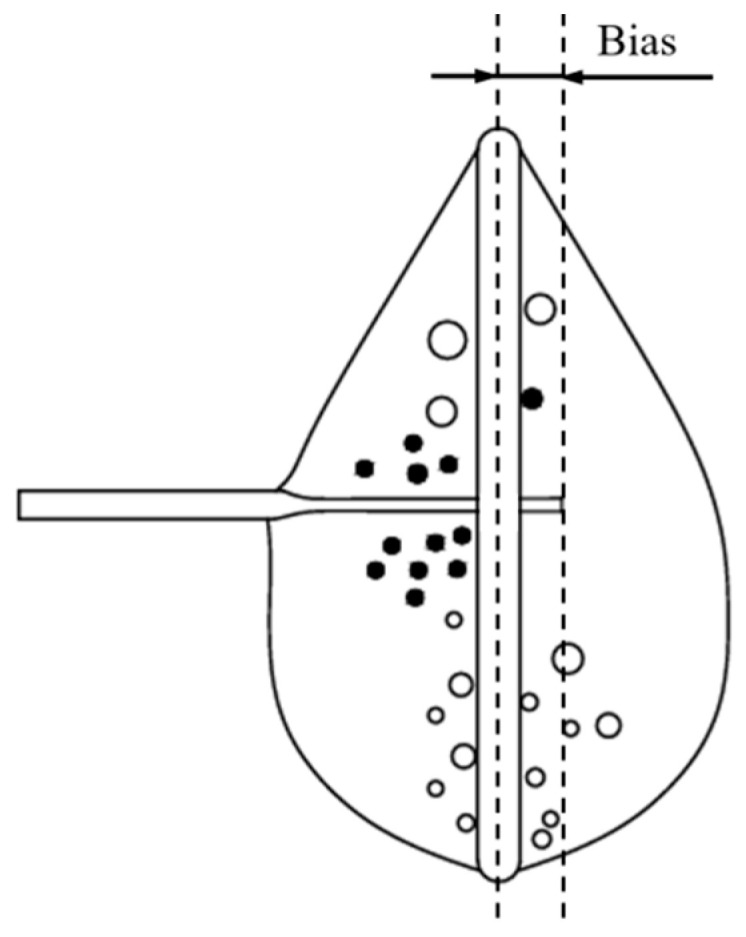
Definition of bias.

For the vertical liquid membrane method, because it prepares microfine tool electrodes by placing the electrode horizontally in the center of the liquid membrane to undergo an electrochemical reaction, the depth of the electrode penetrating into the liquid membrane each time affects the electrode morphology. The distance of the front end of the electrode from the ring surface of the metal ring is defined as the bias, as shown in Figure 4.

**Figure 5:**
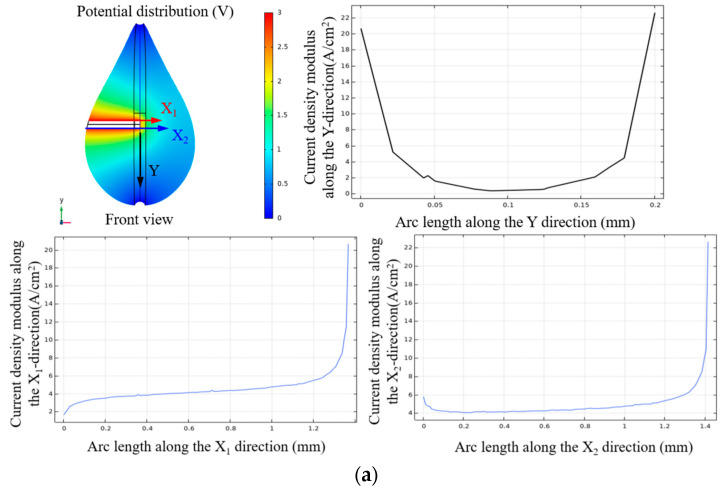

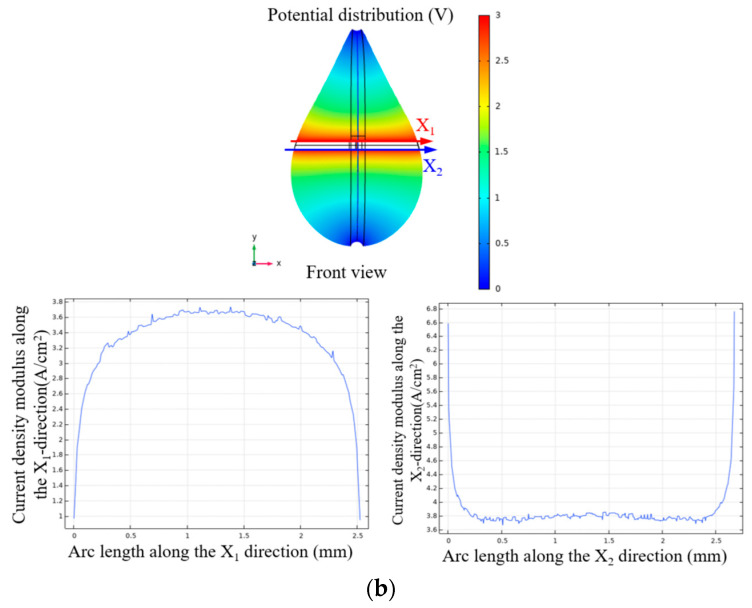
Simulation of current density at the surface of electrodes under different biases in a vertical liquid membrane: (**a**) Bias-0 μm and (**b**) double-ended liquid membrane method.

The simulation results of the vertical liquid film method are shown in Figure 5, where the Y axis in Figure 5a refers to the diameter length range at the front end of the electrode. For the vertical liquid membrane method, single-end and double-end electric field simulation studies are carried out. As shown in Figure 5a, when the electrode is completely located in the liquid membrane, it is found that the current density is maximal at the front end of the electrode and then decreases rapidly in the opposite direction of X_1_ and X_2_; the current density is maximal at the front end of the electrode and then decreases rapidly along the electrode and reaches the minimum at the root of the electrode. In the middle section of the electrode, the current density is more uniformly distributed along the surface of the tungsten rod. In particular, at the front end of the electrode, there is a significant abrupt change in the current density, the value of which is about 4~5 times that of the middle region. When the vertical liquid membrane method is used for processing, the front end of the electrode generates more electrolytic products and wraps around the electrode, which reduces the etching speed of the front-end to some extent. In the middle section of the electrode, the current density is smaller, and fewer electrolytic products are generated. Under the influence of the diffusion layer, the overall etching speed of the electrode can be forced to be regionally uniform, making it possible to prepare micro tool electrodes of uniform size using the vertical liquid membrane method. When the bipolar pulsed vertical liquid membrane method is used for the preparation of micro tool electrodes, the distribution of electrolytic products can be effectively controlled by adjusting the processing parameters to achieve the preparation of tool electrodes with large aspect ratios.

When the electrode passes through the liquid membrane, as shown in Figure 5b, the current density exhibits a symmetrical distribution, and the phenomenon of sudden current changes at the end of the electrodes is improved. In this case, along the X_1_ direction in the diagram, the maximum current density occurs in the middle region of the electrode, while that along the X_2_ direction is located at the boundary of the liquid membrane. This distribution characteristic means that during actual processing, the electrode may first fracture at the boundary of the liquid membrane. The uniform distribution of current density along the electrode, as it passes through the liquid membrane, is advantageous for the preparation of micro tool electrodes with uniform dimensions. However, because the portion of the electrode that passes through the liquid membrane does not participate in the electrochemical reaction, it remains in its original state, affecting the subsequent machining process. Therefore, in order to ensure the quality and functionality of the electrodes, the effects of different parameters on the electrode preparation must be considered comprehensively in the actual processing.

Compared with the single-ended vertical liquid membrane method, the double-ended vertical liquid membrane method has a symmetrical current density distribution, which is uniformly distributed along the surface of the tungsten rods, eliminating the effect of sudden current variations. However, in terms of current density size, the maximum current density of the single-ended vertical liquid membrane method is five times higher than that of the double-ended vertical liquid membrane method.

### 4.2. Effect of Negative Voltage on Electrode Preparation

**Figure 6:**
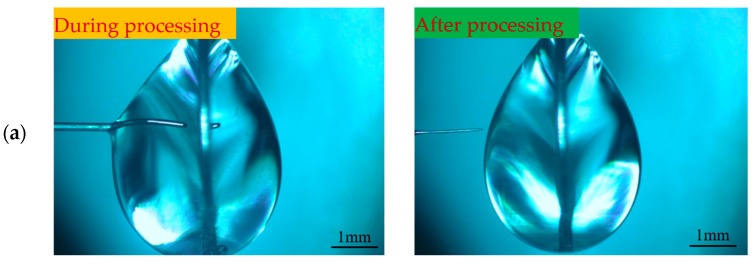

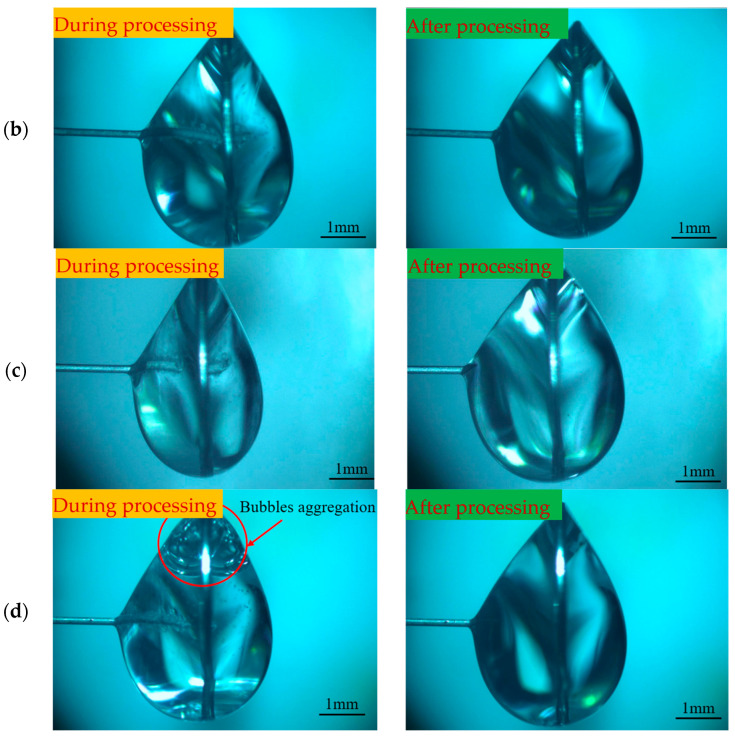
Processing phenomena under different negative pulse voltages: (**a**) −1 V, (**b**) −2 V, (**c**) −3 V, and (**d**) −4 V.

To investigate the effect of different negative voltages on the electrode preparation, a positive voltage of 3 V, a pulse period of 50 μs, a pulse width of 20 μs, an electrolyte concentration of 0.5 mol/L, and negative voltages of −1, −2, and −3 V were selected for the experimental study. Figure 6 shows the processing phenomena observed at different negative voltages. When the negative voltage was small, the hydrogen precipitation current density was not reached, and no hydrogen bubbles were generated on the electrode surface throughout the process. With the increase in negative voltage, the hydrogen generation gradually increased, but the electrode length was significantly shortened. Combined with the electric field simulation analysis, it is inferred that the electrode loses the protection of the diffusion layer under the effect of hydrogen bubble scouring, which exacerbates the etching velocity difference between the tip and the root. Although the shortening of the length of the electrode prepared under negative voltage was not obvious, its processing time was too long, totaling 35 min. To ensure the efficiency and stability of the processing, the negative processing voltage was selected to be −2 V, and the preparation of the tool electrode with a large aspect ratio was investigated under this condition.

### 4.3. Effect of Pulse Width on Electrode Preparation

**Figure 7:**
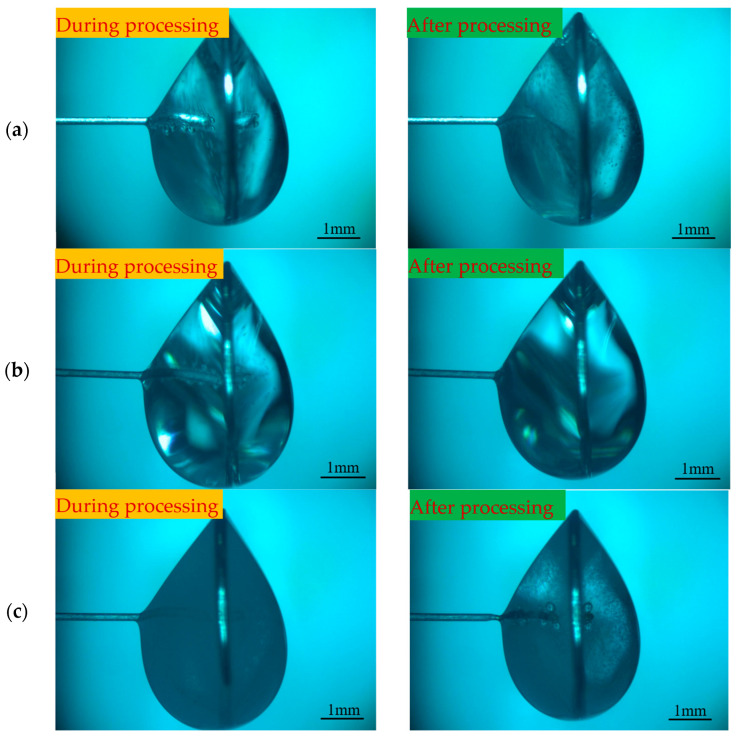

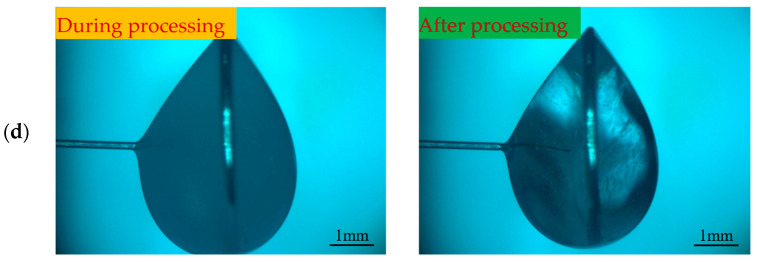
Processing phenomena observed at different pulse widths: (**a**) 10 μs, (**b**) 20 μs, (**c**) 30 μs, and (**d**) 40 μs.

**Figure 8:**
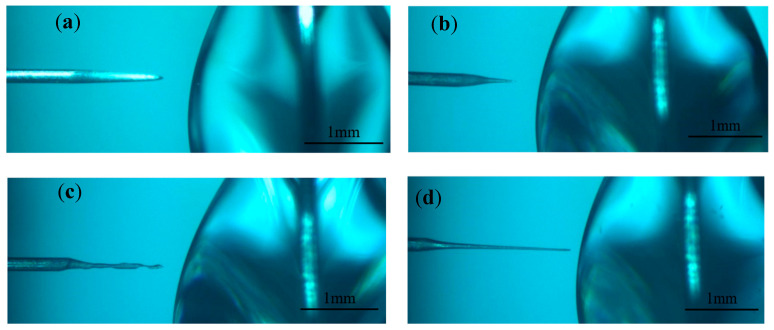
Tool electrodes prepared at different pulse widths: (**a**) 10 μs, (**b**) 20 μs, (**c**) 30 μs, and (**d**) 40 μs.

In order to investigate the effect of different pulse widths on electrode preparation, hydrostatic processing was used, and the positive voltage of 3 V, negative voltage of −2 V, pulse period of 50 μs, electrolyte concentration of 0.5 mol/L, and pulse widths of 10, 20, 30, and 40 μs were selected for the experimental study. Figure 7 shows the processing phenomena observed at different pulse widths. Figure 8 shows the tool electrode prepared after machining.

As the pulse width increased, the hydrogen generation on the metal ring increased dramatically. When the pulse width was less than 30 μs, most of the bubbles within the liquid membrane were generated on the electrode, the electrode surface remains clean throughout the processing, and the electrode was shortened by the etching to the boundary of the liquid membrane in a relatively short time. As seen in Figure 7a, the diameter of the electrode inside the liquid membrane does not change significantly when the pulse width was 10 μs. This is because under the same pulse period, the charging state of the electrode bilayer lasts for a very short time when the pulse width is too small, and the electrode potential only reaches a small value and enters the negative pulse stage, which is far from reaching the decomposition potential of the electrode. In addition, the presence of a negative pulse phase leads to a further decrease in the maximum value that can be reached by the electrode potential in the next pulse phase, which reduces the area in which the electrode reaction occurs. For the vertical liquid membrane method, the value of the current density at the front end of the electrode is much larger than that in other regions due to the sudden change in current density that occurs at the front end of the electrode. Therefore, under low-pulse-width-processing conditions, there is no significant change in the electrode diameter within the liquid membrane. In contrast, the front end of the electrode undergoes a significant etching reaction, and its length is rapidly shortened.

When the pulse width was greater than 30 μs, the positive pulse phase dominated the reaction, the hydrogen generation on the metal ring increased significantly, the liquid membrane was filled with a large number of dense hydrogen bubbles, and the electrode is rapidly etched. When the diameter of the electrode decreased to a certain value, more electrolytic products ${WO}_{3}$ were generated near the electrode per unit time, but due to the exclusion of anions during the negative pulse phase, the ${OH}^{-}$ near the electrode was not enough to react with ${WO}_{3}$ continuously; thus, the electrode surface was covered with insoluble electrolytic products.

Figure 7c,d depict the late processing stage, when the electrode is covered by electrolytic products, the hydrogen bubbles in the liquid film decrease rapidly, and the etching rate is also reduced. Comparing the processing phenomena at pulse widths of 30 μs and 40 μs, although the electrode surface is covered with an electrolytic product cladding layer when the pulse width is 30 μs, a small number of hydrogen bubbles are still produced on the electrode surface due to the negative pulse phase. These bubbles destroy the integrity of the coating layer, resulting in inconsistent etching speeds on all parts of the electrode surface and peculiar shapes of the prepared electrodes. In contrast, under the 40 μs condition, due to the sufficiently large positive pulse width, the amount of electrolytic products generated was large enough to form a dense electrolytic product coating layer, which was difficult to be destroyed by hydrogen. This results in a more uniform distribution of the etching speed across the electrode, and the prepared electrode has a uniform shape and uniform size, as shown in Figure 8d.

Based on the above-mentioned results, controlling the pulse width size is one of the effective ways to solve the problem of rapid reduction in electrode length under bi-directional pulsed power supply conditions and to prepare tool electrodes with large aspect ratios.

### 4.4. Effect of Positive Voltage on Electrode Preparation

**Figure 9:**
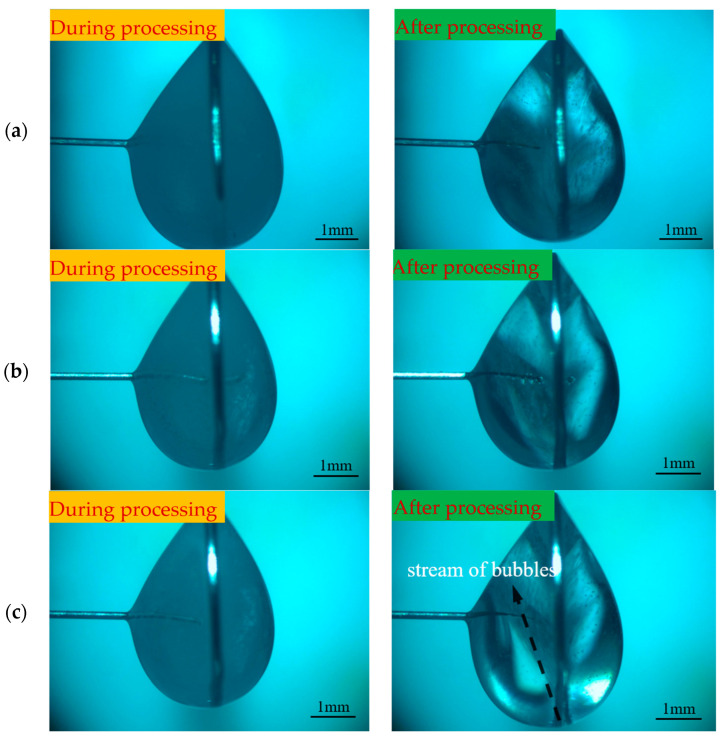
Processing phenomena observed under different positive voltages for large pulse widths: (**a**) 3 V, (**b**) 4 V, (**c**) 5 V.

**Figure 10:**
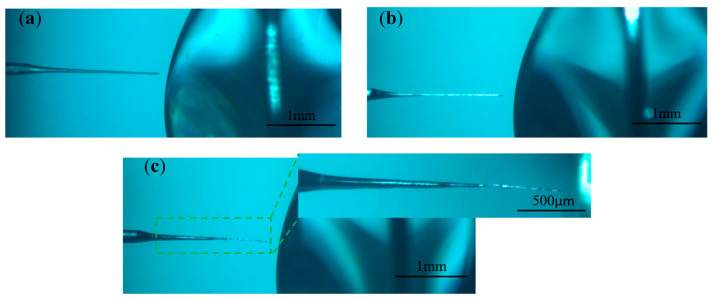
Tool electrodes prepared under different positive voltages for large pulse widths: (**a**) 3 V, (**b**) 4 V, and (**c**) 5 V.

To investigate the effects of different positive voltages on electrode preparation, hydrostatic processing was used. A negative voltage of −2 V, a pulse period of 50 μs, a pulse width of 40 μs, an electrolyte concentration of 0.5 mol/L, and positive voltages of 3, 4, and 5 V were selected for the experimental study. When the positive voltage was small, a large number of hydrogen bubbles were generated on the electrode, which shortened the length of the electrode and destroyed the integrity of the refractory product coating layer. Therefore, the effect of smaller voltage values on the electrode is not considered in this section. Figure 9 shows the processing phenomena observed at different positive voltages. Figure 10 shows the tool electrode prepared after the processing was completed.

By observing the processing phenomenon under large pulse width conditions, the processing stage can be roughly divided into two stages: before the appearance of electrolytic products and after the appearance of electrolytic products. Throughout the processing, the hydrogen precipitation reaction on the electrode in the negative-pulse stage is suppressed due to the large pulse width, and almost no hydrogen is generated on the electrode. Before the appearance of electrolytic products, the liquid membrane is filled with a large number of hydrogen bubbles generated on the metal ring. Due to the excessive number of bubbles, it is difficult to form a stable flow of bubbles; instead, they collide with each other and travel around, so the electrolyte within the liquid membrane is in a state of constant fluctuation, completely breaking the electrolysis products near the electrode. The entire liquid membrane is in dynamic equilibrium with a distribution of the three solutions, electrolysis products, and bubbles. Therefore, in this stage, the electrode diameter decreases rapidly, and its size is more uniform. In this stage, with the increase in positive voltage, the reaction becomes more and more violent, and the electrode diameter also decreases rapidly.

The duration of the above-mentioned stage was then shortened from 10 min to 4 min. When the diameter of the electrode is reduced to a certain degree, the electrolytic product begins to be generated on the surface of the electrode and is tightly encapsulated on the electrode, the hydrogen bubbles within the liquid membrane decrease dramatically, and the etching reaction slows down. The processing phenomenon after the emergence of electrolytic products is observed on the metal ring, and hydrogen generation increases slowly with the increase in the positive voltage. When the positive voltage is less than 5 V, only a small amount of hydrogen bubbles are sporadically generated in the liquid membrane, which does not have a significant impact on the electrolytic product coating layer. When the voltage is increased to 5 V, a bubble stream is formed inside the liquid membrane, which rises inside the liquid membrane along the path shown in Figure 9c and washes over the electrode front end, destroying the coating layer and keeping the surface of the electrode front end clean, which in turn causes the problem of non-uniformity of etching on the electrode. As a result, when the positive voltage is 5 V, the prepared electrode has an extremely thin front end and a thicker root, as shown in Figure 10c. By investigating the effect of different positive voltages on electrode preparation under large pulse width, it was found that large-aspect-ratio tool electrodes with uniform size can be prepared under either 3 or 4 V. However, it takes 30 min to process tool electrodes with the same size under 3 V, but only 12 min under 4 V. Therefore, considering processing efficiency, the positive voltage of 4 V is preferred.

### 4.5. Effect of Bias on Electrode Preparation

**Figure 11:**
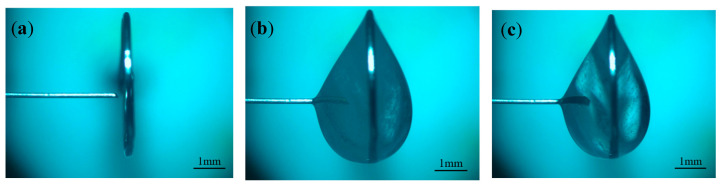

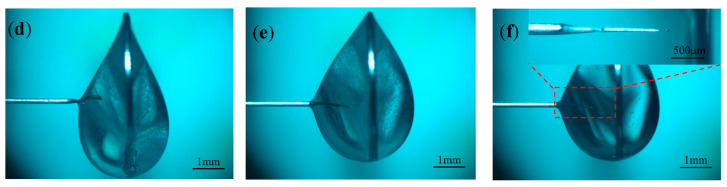
Processing phenomena observed with a bias of −400 μm under large pulse widths (initial state to processing completion): (**a**) The initial state, (**b**) The metal ring produces hydrogen, (**c**) The electrode surface is covered with insoluble electrolyte products, (**d**) The electrode root is abnormally corroded, (**e**) The abnormal corrosion continues, and (**f**) The processing is completed.

**Figure 12:**
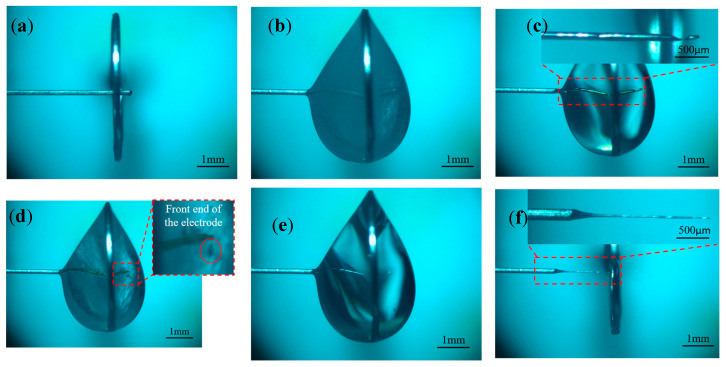
Processing phenomena observed with a bias of 400 μm under large pulse widths (initial state to processing completion): (**a**) The initial state, (**b**) A large number of hydrogen bubbles appears in the liquid membrane, (**c**) Abnormal corrosion occurs at the front end of the electrode, (**d**) The front end of the electrode falls, (**e**) The electrode continues to react and (**f**) The processing is completed.

**Figure 13:**
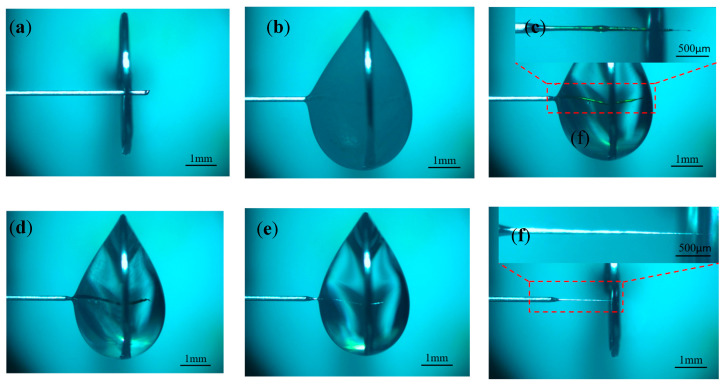
Processing phenomena observed with a bias of 800 μm under large pulse widths (initial state to processing completion): (**a**) The initial state, (**b**) A large number of hydrogen bubbles appears in the liquid membrane, (**c**) Abnormal corrosion occurs at the front end of the electrode, (**d**) The front end is completely corroded, (**e**) The electrode continues to react and (**f**) The processing is completed.

**Figure 14:**
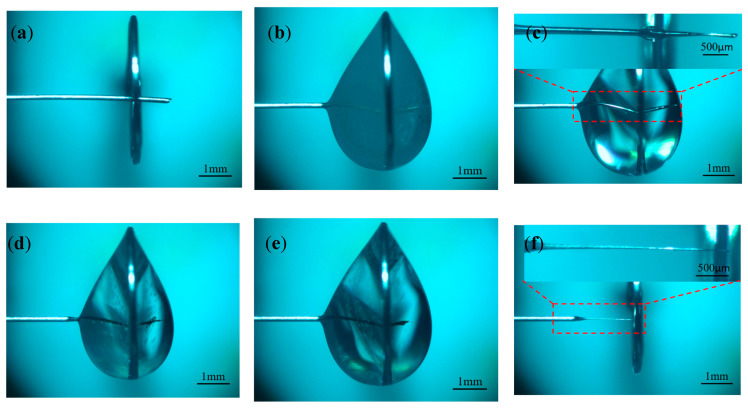
Processing phenomena observed with a bias of 1200 μm under large pulse widths (initial state to processing completion): (**a**) The initial state, (**b**) A large number of hydrogen bubbles appears in the liquid membrane, (**c**) Abnormal corrosion occurs at the front end of the electrode, (**d**) The front end is completely corroded, (**e**) The electrode continues to react and (**f**) The processing is completed.

Under large pulse widths, the problem of inconsistent etching speed at various places of the electrode can be solved by using the insoluble electrolytic products generated on the electrode surface, and then tool electrodes with large aspect ratios can be prepared. To further improve the aspect ratio of the tool electrode, the effect of different biases on the electrode preparation was investigated. Baises of −400, 400, 800, and 1200 μm were selected for the experimental study, and other processing parameters are shown in Table 1. Figure 11, Figure 12, Figure 13 and Figure 14 illustrates the machining phenomena observed at different biases, showing the changes in the morphology of the electrode and the distribution of electrolytic products within the liquid membrane throughout the entire machining process from the initial state to completion.

Comparing the processing phenomena inside the liquid membrane under different bias amounts, when the bias is −400 μm, the electrode is far away from the bubble flow, which avoids the electrolytic product coating layer from being damaged by the impact of the bubble flow. However, because the electrode is immersed in the liquid membrane for a shorter length, the shape of the liquid membrane is more difficult to maintain, and the liquid droplets are easy to fall. Therefore, at the junction of the liquid membrane and the electrode, the surface of the electrode is kept clean by fluctuation in the liquid membrane, and the etching speed is instead greater than that of the other regions, resulting in the electrodes prepared under the negative bias amount having a finer root and a coarser middle, as shown in Figure 11f.

When the electrode bias is positive and all of them are located inside the liquid membrane, many hydrogen bubbles are generated on the metal ring. However, the shape of the liquid membrane on both sides of the metal ring is not exactly the same, resulting in hydrogen bubbles appearing first on the right side of the liquid membrane and then diffusing into the liquid membrane. As a result, the electrode located on the right side of the liquid membrane is continuously impacted, resulting in abnormal corrosion, as shown in Figure 12, which may produce notches on the electrode neck or result in the front of the electrode having a significantly smaller diameter than the other areas. As the processing continues, the diameter of the electrode neck continues to decrease, and at some point, the front end of the electrode breaks off at the neck, as shown in Figure 12d, where a small section of the electrode front end can be observed to fall off. Thereafter, the electrode surface is completely covered by electrolytic products, the bubble generation decreases dramatically, and the etching speed of each region of the electrode tends to be consistent. When the bias amount is 0 μm, as shown in Figure 9b and Figure 10b, the electrode is not affected by the bubble flow on the right side, and the electrode surface can be completely covered by the electrolytic product until the processing is completed without causing abnormal corrosion.

**Figure 15:**
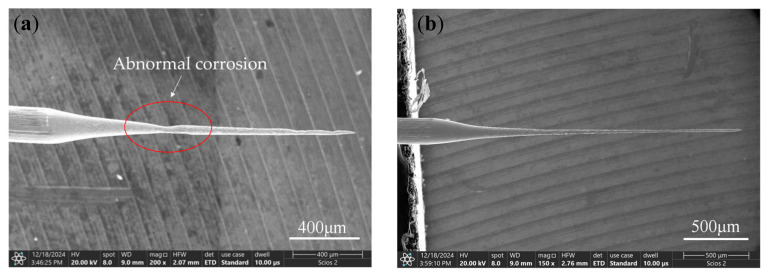

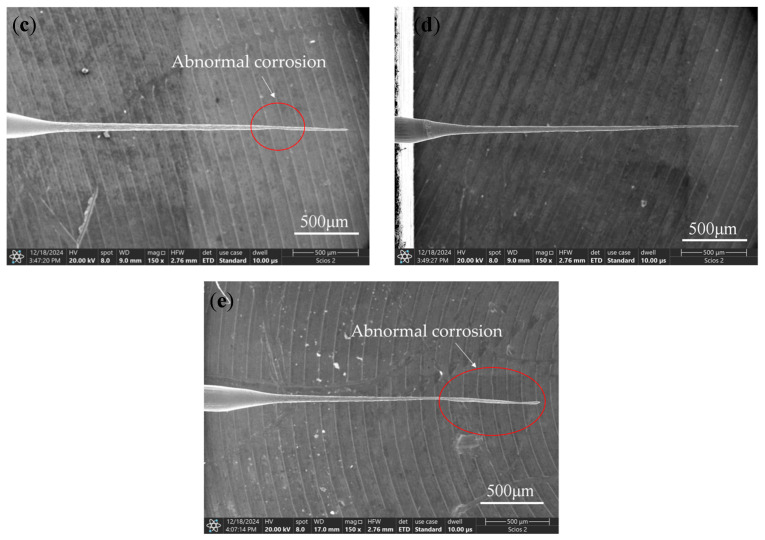
Tool electrodes prepared under different biases at large pulse widths: (**a**) −400 μm, (**b**) 0 μm, (**c**) 400 μm, (**d**) 800 μm, and (**e**) 1200 μm.

**Figure 16:**
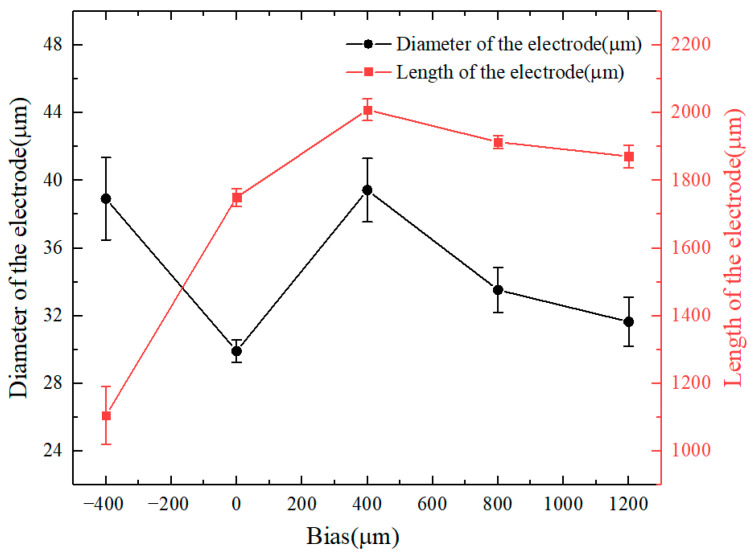
Trend plot of electrode diameter and length with bias change. The error bars represent ± SD from the mean value (n = 3).

Figure 15 shows the microfine tool electrodes prepared under different bias amounts. Figure 16 shows the trends in electrode diameter and length with the change in bias. With the increase in bias, the electrode diameter is roughly around 35 μm and the electrode length gradually increases. When the bias is negative, abnormal corrosion occurs at the root of the electrode, which makes it difficult to prepare an electrode with uniform size. When the bias is positive, the front end of the electrode is affected by bubbles, resulting in abnormal corrosion, which is not conducive to the stability of the process. When the bias is zero, the micro tool electrode with uniform shape can be prepared more stably, as shown in Figure 15b, the average diameter of the microfine tool electrode with zero bias is about 28 μm, the length of the electrode is 1570 μm, and the length-to-diameter ratio is 56.1.

To solve the problem of abnormal corrosion at the front end of the electrode caused by the inconsistent shape of the liquid membrane on both sides of the metal ring under positive bias, and to further improve the electrode aspect ratio, the distribution of electrolytic products within the liquid membrane when the electrode passes through the liquid membrane and its influence on the preparation of the electrode were investigated.

**Figure 17:**
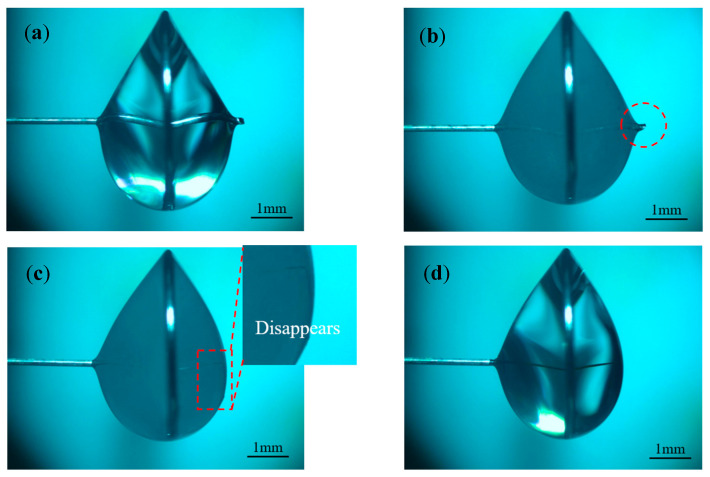
Processing phenomena observed when the electrode happens to penetrate the liquid membrane: (**a**) initial state, (**b**) during processing, (**c**) electrode front end bent and broken, and (**d**) electrode surface coated with electrolytic products.

**Figure 18:**
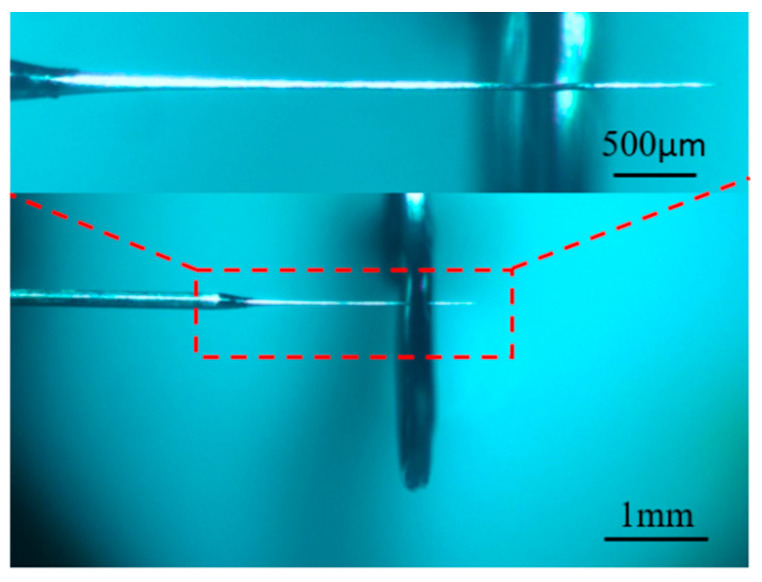
Shape of the electrode after processing.

The processing phenomenon observed when the electrode is exactly across the liquid membrane is shown in Figure 17. The liquid membrane on both sides of the metal ring has the same shape, which makes the current density on both sides of the electrode and the distribution of bubble flow within the liquid membrane symmetrical. Due to the fast etching rate of the electrode at an electrolyte concentration of 0.5 mol/L, hydrogen bubbles are spread in the liquid membrane, and the electrolytic products on the surface of the electrode are completely dispersed, so the electrode is slim and uniform in size before the appearance of electrolytic products. When the electrode size is reduced to a certain extent, the electrode inside the liquid membrane is affected by the gravity of the electrode, which penetrates the liquid membrane on the right side. This penetration causes the front end of the electrode to bend or break, as shown in Figure 17c. Subsequently, the electrode surface is rapidly enveloped by insoluble products, as shown in Figure 17d. When processed for a period of time, the insoluble electrolytic products on the surface can be dispersed to obtain the electrode of the desired diameter, as shown in Figure 18. By controlling the length of the electrode through the liquid membrane, it is possible to control the location of the front end of the electrode where the fracture occurs, reduce the loss of the electrode, and improve the electrode aspect ratio.

### 4.6. Preparation of Micro Tool Electrodes with Large Aspect Ratios

**Figure 19:**
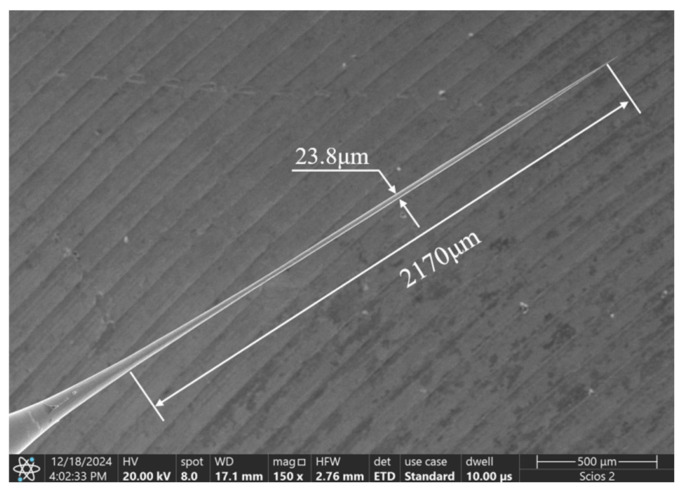
Tool electrodes prepared at exactly the time of penetration into the liquid membrane.

Under the processing conditions of positive voltage of 4 V, negative voltage of −2 V, pulse width of 40 μs, pulse period of 50 μs, and the electrode crossing the liquid film, the tool electrode with a large aspect ratio was finally prepared. Figure 19 shows the tool electrode prepared when the electrode passes through the liquid membrane; the average diameter is approximately 23.8 μm, the length of the electrode is 2170 μm, and the aspect ratio is 91.2. Compared with the electrodes prepared under zero bias, the aspect ratio of the tool electrode prepared by passing through the liquid membrane is much larger than that prepared under zero bias, even though the tool electrode loses a part of its length.

## 5. Conclusions

To achieve efficient preparation of large-aspect-ratio tool electrodes, a bipolar pulsed vertical liquid film electrochemical etching method was proposed in this paper. An electric field simulation analysis of single-ended and double-ended vertical liquid membrane method was carried out, and the effects of negative voltage, pulse width, positive voltage, and bias on the distribution of electrolytic products and electrode preparation were investigated. The following conclusions were drawn:

1. A bipolar pulsed vertical liquid film electrochemical etching method was proposed. It was found that the introduction of negative voltage would generate a large amount of hydrogen on the electrode surface, wash away the diffusion layer, and have a significant effect on the movement of electrolytic products.
2. For the single-ended vertical liquid membrane method, the current density is maximal at the tip of the electrode and decreases along the tungsten rod towards the root. For the double-ended vertical liquid membrane method, the current density is symmetrically distributed, which is conducive to the preparation of column electrodes with uniform size.
3. With the increase in negative voltage, the amount of hydrogen generation increases and the generation of electrolytic products on the processed surface decreases significantly, but the electrode length shortens significantly. With the increase in pulse width, the amount of hydrogen generation decreases, the surface gradually generates insoluble electrolytic products, and the phenomenon of electrode length shortening is alleviated.
4. Under large pulse width, with the increase in positive voltage, the surface of the electrode gradually generates a dense insoluble product coating layer, which is conducive to the preparation of the column electrode with a uniform size and large aspect ratio.
5. Under the processing conditions of large pulse width, when the single-ended vertical liquid membrane method is used, the electrode diameter is roughly 35 μm, whereas the electrode length becomes gradually larger with the increase in the bias amount. However, when the bias amount is greater than zero, the front end of the electrode is prone to abnormal corrosion.
6. When the front end of the electrode happened to penetrate the liquid membrane on the right side, a large aspect ratio tool electrode with an average diameter of about 23.8 μm, an electrode length of 2170 μm, and an aspect ratio of 91.2 was prepared by losing a small amount of the electrode that penetrated the liquid membrane. The corresponding processing parameters are a positive voltage of 4 V, negative voltage of −2 V, pulse width of 40 microseconds, and pulse period of 50 microseconds.

However, when the micro electrode is prepared by the bipolar pulsed vertical liquid film method, the aspect ratio and shape of the prepared electrode will be affected by the distribution of the insoluble electrolyte layer. Therefore, it is necessary to further study the specific effects of the movement of anions and cations on the distribution of insoluble electrolytes and analyze the relationship between the change in electrode diameter and the insoluble electrolyte layer, so as to realize the online preparation of micro-tool electrodes.

## Figures and Tables

**Table 1:** Processing parameters of the test.

Parameter	Value
Positive voltage $U_{p}\;$[V]	4
$\mathrm{Negative}\;\mathrm{voltage}\;U_{n}\;$[V]	−2
Pulse period T [μs]	50
Positive pulse width $t_{p}\;$[μs]	40
Electrolyte concentration [mol/L]	0.5
Initial diameter of the electrode [μm]	200

## Data Availability

The original contributions presented in this study are included in the article. Further inquiries can be directed to the corresponding author.
